# Chemoimmunotherapy in Advanced, PD-1 Refractory Cutaneous Squamous Cell Carcinoma: Insights From Two Immunocompromised Patient Cases

**DOI:** 10.1155/crom/4443916

**Published:** 2025-07-14

**Authors:** Golbarg Rahimi, Gino K. In

**Affiliations:** ^1^Keck School of Medicine, University of Southern California, Los Angeles, California, USA; ^2^Norris Comprehensive Cancer Center, University of Southern California, Los Angeles, California, USA

## Abstract

Cutaneous squamous cell carcinoma (cSCC) is one of the most prevalent malignancies worldwide, and a subset of patients, particularly immunocompromised individuals, will face heightened risks of metastasis and mortality. This report examines two cases of immunocompromised patients treated with a combination of platinum- and taxane-based chemotherapy together with PD-1 inhibition, having progressed after PD-1 monotherapy. The first patient, a 54-year-old man with HIV and recurrent metastatic cSCC, had rapid progression of disease while undergoing PD-1 inhibition with cemiplimab but later achieved tumor control when treated with pembrolizumab, carboplatin, and paclitaxel. The second patient, a 36-year-old man with cystic fibrosis and a history of lung transplantation, had no signs of response to cemiplimab but experienced a partial response when treated with cemiplimab, carboplatin, and paclitaxel. These cases suggest that combining chemotherapy with PD-1 inhibition may help overcome primary resistance to PD-1 therapy in advanced cSCC.

## 1. Introduction

Cutaneous squamous cell carcinoma's (cSCC) health burden is often underestimated among the general population [1]. Nevertheless, it accounts for 20% of all nonmelanoma skin cancers and has the ability to spread to various organs and result in death [2]. The metastatic rate for cSCC is 1.9%–2.6% over a median follow-up of 70 months, with most metastases detected within the first 2 years after diagnosis [3], demonstrating the potential for aggressive nature in a subset of these patients [4]. In some regions of the United States, mortality rates from cSCC may be as high as those of renal and oropharyngeal carcinomas, as well as melanoma [1]. Immunocompromised patients, including those with solid organ transplantation, hematologic malignancies, chronic viral infections, and autoimmune disease, have greater risk of metastases and death from cSCC [5, 6].

Treatment options for cSCC, particularly when it recurs or metastasizes, are unfortunately limited. Immune checkpoint inhibitors (ICI) that target the PD-1/PD-L1 axis are the primary systemic treatment approach [7], and the only FDA-approved therapy for cSCC. ICI may lead to durable responses as well as complete responses for patients with recurrent or metastatic cSCC, but only roughly 50% of patients will achieve a response to treatment; for those who do not respond to this therapy, other systemic options include cytotoxic chemotherapy or EGFR inhibition. Importantly, cytotoxic chemotherapy (i.e., platinums and taxanes) may enhance the effectiveness of ICI by upregulating PD-L1 expression on cancer cells and by promoting antitumor immunogenicity [8]. Accordingly, chemoimmunotherapy (CIO), the use of these agents together, has been utilized with synergistic effects and led to improved clinical outcomes in multiple tumor types, including lung [9], breast [10, 11], and cervical cancers [12]. This case report describes two different immunocompromised cSCC patients who did not respond to PD-1 therapy alone, but later benefitted from this CIO combination strategy.

## 2. Methods

Ethical approval for this retrospective study was granted by the Institutional Review Board (IRB). Informed consent was not obtained as the participants are deceased. However, no identifying information was included, ensuring that patient confidentiality is maintained. Given the nature of the study, with no direct interaction with living individuals and no harm to the deceased participants, the study was conducted in compliance with ethical guidelines.

## 3. Patient One

The first patient was a 54-year-old gentleman with a history of HIV and hepatitis B coinfection on HAART (last CD4 count 897, undetectable VL) who presented in 2008 with a cSCC of the right great toe requiring amputation. The patient then presented with a locoregional, cutaneous metastasis at the right second toe 11 years later in 2019, requiring a partial amputation. There was a second recurrence of locoregional cSCC at the base of the right second amputation site 8 months later, for which complete amputation was then performed. In 2020, the patient experienced progression of disease in the right lower extremity, with multiple in-transit cutaneous metastases involving the right upper thigh, right shin, and right knee, as well as multiple right inguinal lymph nodes, with multiple biopsies confirming metastatic cSCC. Given the extent of disease, systemic therapy was recommended, and the patient underwent 3 months of PD-1 inhibition with cemiplimab from November 2020 to February 2021; there was no clinical or radiographic sign of response. In March 2021, the patient underwent palliative resection of multiple cSCC lesions, including the right distal foot, the right anterior proximal foot, the right anterior shin, the right medial calf, right medial knee, right groin, and right medial thigh. Unfortunately, just 1 month later, the patient developed rapid progression again within the right lower extremity, including multiple in-transit cutaneous and subcutaneous lesions and regional lymph nodes. The patient was then treated with 3 months of chemotherapy and EGFR inhibition, using capecitabine, carboplatin, and cetuximab; however, follow-up imaging showed disease progression. The patient was then treated on a clinical trial using a monoclonal antibody targeting caprin-1 with topical imiquimod, but again had progression of disease. Molecular testing showed no pathogenic mutations, low TMB (2 mut/Mb), and 2% PD-L1 expression.

As no actionable targets were identified, in October 2021, the patient was started on a CIO combination, using pembrolizumab 200 mg, carboplatin AUC 3, and paclitaxel 120 mg/m2, every 3 weeks. Repeat imaging in December 2021 showed a response to treatment with decreased size of multiple lymph nodes and in-transit/cutaneous metastases; the patient noted clinical benefit with less pain and bleeding from multiple tumor sites. Follow-up scans in April 2022 demonstrated disease progression with a new right 7th rib mass with associated pathologic fracture; this lesion was treated with palliative radiation. The patient continued to receive a total of 14 cycles of this combination therapy for nearly 10 months before there was eventual progression of disease in September 2022, when the patient presented with a progressive right anterior tibial mass with associated pathologic fracture, requiring hip disarticulation. The patient continued to have progressive disease and sought second opinions, but ultimately passed away due to the underlying malignancy in November 2023.

## 4. Patient Two

The second patient was a 36-year-old gentleman with a history of cystic fibrosis who underwent lung transplantation in 2006, thus requiring chronic immune suppression to prevent graft rejection. In December 2018, he was diagnosed with a cSCC of the right nasal ala, which required Mohs surgery. In December 2019, he presented with recurrent disease at the resection site, and again underwent Mohs surgery. He then completed a total of 60 Gy/30 fx adjuvant radiation to the nasal ala. In September 2020, the patient developed a second recurrence, this time at the upper right nasolabial fold and right upper lip, consistent with multiple locoregional, cutaneous metastases. In October 2020, the patient started therapy with the EGFR inhibitor cetuximab; there was no obvious benefit from treatment, and CT scan in December 2020 showed regional metastasis to a right submandibular lymph node. The patient underwent right modified radical neck dissection to Levels IB/IIA/IIB/III/IV, as well as resection of the nasolabial mass, right upper lip mass, and separate soft tissue face mass; pathology showed a poorly differentiated squamous cell carcinoma with sarcomatoid features, perineural invasion, and 3 out of 32 nodes involved. The patient underwent adjuvant radiation, 66 Gy over 30 fractions to the right neck; concurrent capecitabine was attempted but not well tolerated due to fatigue, anorexia, nausea, and renal insufficiency and, thus, was stopped after 3 weeks.

After a 3-month break from therapy, the patient had a third recurrence to the right nasal bridge in June 2021; PET scan at the time also showed a 9-mm left cervical 1B lymph node. One month later, the patient underwent left modified radical neck dissection of Levels IB/IIA/III, partial rhinectomy, and right medial canthoplasty; pathology revealed a 3.5-cm cSCC of the nasal bridge, also additional disease involving the right cheek, right upper lip, and nasal dorsum and involving 3 out of 16 lymph nodes. A fourth locoregional recurrence to the right face, nose, and right nasolabial fold occurred just 3 months later; this also required additional surgery, with a right face lesion excision, total rhinectomy, and right lip wedge excision, and pathology again showed recurrent cSCC. The patient then underwent adjuvant radiation, 200 cGy × 33 fractions, to the left neck Levels IB–IV.

Following a fifth locoregional recurrence to the right nasal bridge, right cheek, and right nasolabial fold, the patient was treated with three cycles of intratumoral therapy using TVEC (talimogene laherparepvec) in December 2021. The patient experienced clear clinical progression of disease, with the tumor causing severe ulceration with an open wound into the right side of the face and approaching the right eye. Tumor molecular profiling showed TMB of 17 Mut/Mb, in addition to CDKN2A, FANCA, TP53, and STK11 mutations. PD-L1 expression was not reported. From February 2022–March 2022, the patient was started on PD-1 inhibition with cemiplimab, together with prednisone and tacrolimus to prevent graft rejection; there were no signs of clinical or radiographic response. From June 2022, the patient was then treated with a CIO combination of cemiplimab 350 mg IV every 3 weeks and weekly carboplatin AUC 2 and paclitaxel 60 mg/m2. After 3 weeks of treatment, the patient reported clinical benefit with decreased pain and reduction of a tumor on the right cheek. After 6 weeks, treatment was complicated by neutropenic fever, requiring dose reduction, but the patient was eventually able to resume treatment. A PET scan from September 22 showed a partial response to treatment, with decreased metabolic activity of a prominent right face/cheek mass (SUVmax 3.9 down from 9.9). The patient was continued on therapy for another 3 months until December 2022, but given persistent neutropenia, as well as the clinical response to treatment, the decision was made to continue with cemiplimab alone. In January 2023, the patient was hospitalized with hypoxic respiratory failure, attributed to PD-1 induced pneumonitis; despite aggressive management, the patient unfortunately had progressive respiratory failure and was transitioned to comfort care.

## 5. Discussion

Advanced cSCC continues to be a complex and challenging scenario for clinicians. Recently, ICI, which block the PD-1/PD-L1 pathway, have led to improved outcomes for patients with advanced cSCC, most notably with durable responses and low rates of toxicity [7]. Aside from these, though, there are few other systemic options, and this remains a critical unmet need for the roughly 50% of cSCC patients who will not benefit from ICI. While cytotoxic chemotherapy and EGFR inhibitors have been historically used to treat cSCC, neither of these approaches achieves durable disease control, and the role of these agents in the PD-1 failed setting, in particular, remains unclear. There are ongoing efforts to study the combination of EGFR inhibitors together with ICI (NCT03944941), and meanwhile, previous studies have shown that the combination of cytotoxic chemotherapy and ICI is effective for other cancer types [9, 11, 12]. As such, we sought to explore this CIO approach for cSCC.

Here, we describe two different patients with advanced, refractory cSCC that were not responsive to single-agent PD-1 inhibition, but later responded to a CIO combination of PD-1 with carboplatin and paclitaxel. Both of these patients had previously experienced recurrent disease despite multiple surgeries (one also with radiation) and also were not responsive to multiple systemic treatments, including cytotoxic chemotherapy and EGFR inhibitors; thus, these patients had very limited remaining options. Of note, molecular testing did not reveal any obvious indication of resistance mechanisms for either patient, neither to PD-1 inhibition nor EGFR inhibition.

Therefore, it is quite intriguing that in such heavily pretreated patients, CIO would result in clear clinical benefit. Indeed, whereas both patients had been exposed separately to ICI and chemotherapy, it was only when these two approaches were used together that a treatment response occurred. Furthermore, it is also notable that these responses occurred in patients with underlying immunosuppressed states, where one would expect less robust immune responses. Unfortunately, both responses were short-lived, one patient having progressive disease after 6 months, and the second patient having grade 4 toxicity from therapy after 7 months. Still, to the best of our knowledge, these two patient examples provide the first reported cases of salvage CIO for PD-1 refractory cSCC. As noted earlier, this regimen has proven effective in other tumor types, where PD-1 monotherapy may not be as effective as CIO. Further research is essential to confirm the efficacy and safety of combining chemotherapy with ICI for cSCC.

## Data Availability

The data that support the findings of this study are available on request from the corresponding author. The data are not publicly available due to privacy or ethical restrictions.
